# Processing of Thermotropic Fully Aromatic Polyesters by Powder Molding Accompanied by Solid-State Post-Polymerization

**DOI:** 10.3390/polym17101358

**Published:** 2025-05-15

**Authors:** Pavel A. Mikhaylov, Anton V. Mityukov, Dmitry V. Dudka, Yaroslav V. Golubev, Valery G. Kulichikhin, Alexander Ya. Malkin

**Affiliations:** A. V. Topchiev Institute of Petrochemical Synthesis, Russian Academy of Sciences (TIPS RAS), 29 Leninsky Prospekt, 119991 Moscow, Russia; ant-mityukov@yandex.ru (A.V.M.); dudka@ips.ac.ru (D.V.D.); ya_golubev@ips.ac.ru (Y.V.G.); klch@ips.ac.ru (V.G.K.); alex_malkin@mig.phys.msu.ru (A.Y.M.)

**Keywords:** thermotropic polyesters, solid-state polymerization, liquid crystal phase, injection molding, titanium dioxide

## Abstract

Thermotropic polyesters are a subject of keen interest due to their exceptional heat resistance, thermal stability, and high strength. However, these thermal characteristics pose significant constraints on standard manufacturing processes, as the melting temperatures of these polymers can exceed 300 °C. This study explored the feasibility of manufacturing final items molded from prepolymers through a solid-state polymerization process. A copolymer composed of 4-acetoxybenzoic acid (4ABA), 3-acetoxybenzoic acid (3ABA), and 4′-acetoxybiphenyl-4-carboxylic acid (ABCA) was synthesized using melt polycondensation. To comprehensively evaluate the performance of the resulting material, several sets of samples were prepared, including those containing TiO_2_. Experimental samples from the pre-polymers were obtained through injection molding followed by high-temperature solid-state post-polymerization. The final products underwent a range of tests, including rheological and mechanical analyses, as well as thermal evaluations. The products demonstrated sufficient strength and stability. The proposed method of solid-state post-condensation offers significant potential advantages for the practical application of manufacturing high-performance engineering materials.

## 1. Introduction

Thermotropic main-chain polyesters (TMCPs) are a family of high-performance polymers characterized by a unique set of properties that allow the production of high-strength fibers and molded products with excellent heat resistance and thermal stability. [1,2,3,4,5,6,7]. The synthesis and processing of TMCPs do not require large amounts of solvents, which is in line with the principles of green chemistry. The family of TMCPs includes semi-aromatic polyesters, which consist of aromatic units alternated with flexible alkyl chains in their main chain, and fully aromatic copolyesters, which consist of various types of rigid aromatic units. The latter exhibit significantly higher chemical and thermal stability (up to 500 °C in an inert atmosphere) and heat resistance (with a heat bending temperature exceeding 300 °C) [8]. Due to their high chemical stability, fully aromatic TMCPs are not considered biodegradable polymers. However, they can be degraded through methanolysis in the presence of a base or superbase [9,10]. To date, numerous fully aromatic TMCPs have been described in scientific papers and patents. Typically, these fully aromatic TMCPs are random copolyesters comprising rigid aromatic mesogenic and non-mesogenic units, as well as aromatic units with aliphatic and aromatic side chain groups supported by the copolymerization of several comonomers [11,12,13,14]. The copolymerization of several types of aromatic comonomers has been described, which includes a certain amount of non-mesogenic, so-called “kinked” comonomers, which help to lower the melting point and enhance solubility while preserving the liquid crystalline state of the copolyesters [5,6,10].

The most well-known industrially produced fully aromatic TMCPs are Vectra A-950, a copolyester composed of 4-hydroxybenzoic acid (4HBA) and 6-hydroxy-2-naphthoic acid [4,7,15] as well as Ekonol and Xydar, which consist of 4HBA, 4,4′-biphenyldiol, terephthalic acid, and isophthalic acid units [4,7,16,17]. However, the synthesis of fully aromatic TMCPs requires harsh polymerization and processing conditions, with temperatures reaching up to 400 °C [18]. In addition, the viscosity of high-molecular-weight fully aromatic TMCPs is excessively high, requiring specialized equipment and increasing energy consumption.

Several patents describe a two-step method of synthesis aromatic TMCPs [19,20,21,22]. In the first step, low-molecular-weight prepolymers are produced. These prepolymers are then ground and subjected to solid-state polymerization (SSP) to produce advanced polymers. In addition, the advanced polymers can be used to produce fibers or be melt-blended with fillers to produce injection-molded compounds. This approach eliminates the need to mix a highly viscous reaction mixture in the first step. However, melt-mixing with fillers or processing advanced polymers can be problematic due to their high viscosity and elevated processing temperatures. In contrast, prepolymers have low viscosity and can be processed at lower temperatures. Moreover, the low viscosity of prepolymers allows for more random distributing filler particles within the matrix. Thus, blending low-viscosity prepolymers with fillers appears to be a promising method for producing final products that can subsequently be subjected to solid-state post-polymerization.

In our previous work [2], we synthesized a novel TMCP based on 4-hydroxybenzoic acid (4HBA), 3-hydroxybenzoic acid (3HBA), and 4′-hydroxydiphenyl-4-carboxylic acid (HBCA). The aim of the present study was to investigate the optimal process for producing both filled and unfilled samples from the TMCP prepolymer, focusing on achieving the best mechanical properties while controlling porosity. We conducted pilot-scale melt polycondensation of the low-molecular-weight copolyester (pre-polymer) described in our previous work [2]. Molded articles, both filled and unfilled, were produced from the copolyester using a powder molding technique followed by solid-state polymerization for various periods of time. The molecular weight changes of the copolyester were monitored using rotational rheology and intrinsic viscosity measurements.

## 2. Materials and Methods

### 2.1. Materials

4-Hydroxybenzoic acid (4HBA, >98% purity) was produced by Acros Organics (Mumbai, India). 3-Hydroxybenzoic acid (3HBA, >98% purity) was produced by Reachem (Moscow, Russia). DMSO-*d*_6_, methanol-*d*_4_, and chloroform-*d* (>99.8% purity) were purchased from Cambridge Isotope Laboratories (Tewksbury, MA, USA). Trifluoroacetic acid (TFA, >98% purity) was offered by Fluka (Buch, Switzerland). All other solvents (analytical grade) were purchased from Ekos-1 LLC (Moscow oblast, Staraya Kupavna, Russia).

4-Acetoxybenzoic (4ABA) and 3-acetoxybenzoic (3ABA) acids were prepared by the refluxing (2 h) of in toluene with 20% molar excess of acetic anhydride according previous studies [10,23].

4′-Acetoxybiphenyl-4-carboxylic acid (ABCA) was synthesized at Yaroslavl State Technical University (Yaroslavl, Russia). For ABCA: ^1^H NMR (400 MHz, CDCl_3_) δ, ppm—8.30 (t, J = 1.8 Hz, 1H), 8.10 (dt, J = 7.9, 1.4 Hz, 1H), 7.86 (dt, J = 8.0, 1.4 Hz, 1H), 7.68–7.62 (m, 2H), 7.58 (t, J = 7.8 Hz, 1H), 7.23–7.17 (m, 2H), 2.45 (s, 3H).

**Figure 1:**
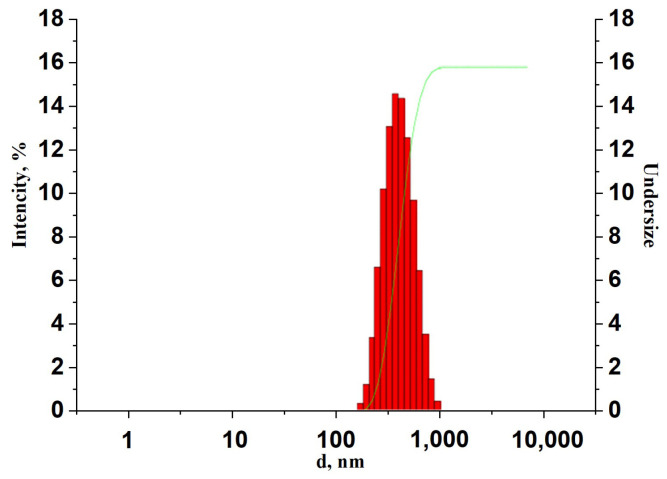
Size distribution of Titanium dioxide Ti-Pure^TM^ R-900.

Titanium dioxide Ti-PureTM R-900 (The Chemours Company, Wilmington, DE, USA) with an average size distribution around 400 nm was used for compound preparation. The measurement of the size distribution was performed by dynamic light scattering in a Zetasizer Nano-ZS (Malvern Panalytical, Malvern, UK) analyzer at a scattering angle of 173°. The data were averaged over 10 separate measurements for each sample. The experiments were carried out for dilute suspensions at a concentration of 0.05 wt.%. The TiO_2_ size distribution diagram is shown in Figure 1.

### 2.2. Prepolymer Synthesis

178.62 g of 4ABA (0.99 mol, 70% mol), 25.52 g of 3ABA (0.142 mol. 10% mol), and 72.58 g of ABCA (0.284 mmol, 20% mol) were loaded into a LIST CRP 2,5 B 4.6-L twin-shaft reactor equipped with a Lauda heating unit (LIST Technology AG, Arisdorf, Switzerland), an inert gas inlet, and a condenser. The reactor was gradually heated to 212 °C under a slow flow of argon. During this stage, no evolutions of volatile polycondensation products were observed. The temperature was then increased to 273–274 °C over 150 min, reaching the apparatus’ temperature limit, and maintained for an additional 30 min. Upon cooling, 136 g of the prepolymer (designated as SI) was extracted from the reactor. The resulting prepolymer was brittle, with an intrinsic viscosity as low as 0.5. The prepolymer was ground into small pieces, loaded into a tube furnace, and heated from room temperature to 170 °C for approximately 1 h under a stream of argon. Subsequently, it was gradually heated over 4 h to 240 °C. The prepolymer was then held at 240 °C, 250 °C, and 260 °C for 2 h at each temperature. The treated prepolymer, with an intrinsic viscosity of 3.1, was designated as ST.

### 2.3. Sample Preparation

**Figure 2:**
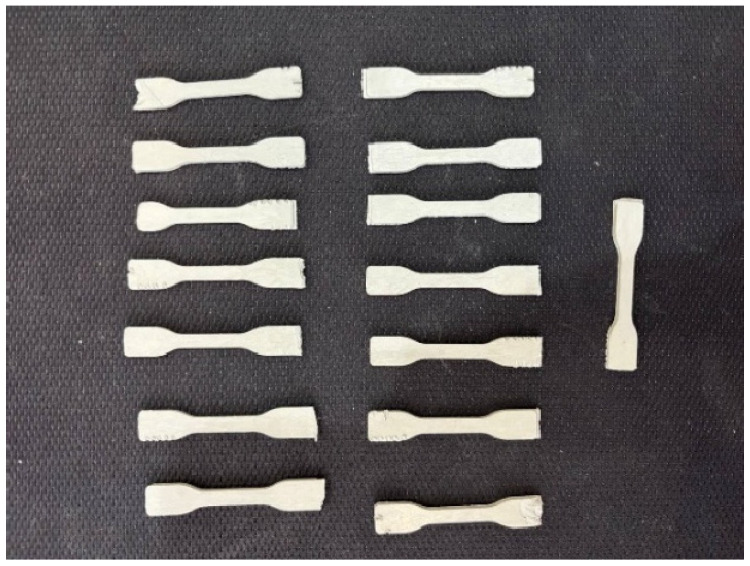
Samples prepared via injection molding.

Injection molding for sample preparation was performed using the IM 12 Xplore (Sittard, The Netherlands) injection molding machine according to the following protocol. Powder, pre-dried for 3 h at 120 °C, was loaded into a chamber heated to 320 °C and injected into the mold, heated to 100 °C, at a pressure of 8 bar. After air-cooling to room temperature, the specimens were manually removed from the molds. The shape of the samples conformed to the international standard [24] for determining the strength properties of plastics (Figure 2). Fifteen specimens were injected for each series of tests and were mechanically labeled (marks 1 to 15) for further determination of mass loss and density after solid-state post-polymerization.

### 2.4. Polymer Compounding

A compound with TiO_2_ 30 wt.% content was prepared in a HAAKE PolyDrive (Karlsruhe, Germany) rotary mixer according to the following protocol: preliminary crushed ST prepolymer was loaded into the mixer chamber, heated at 320 °C. Then, after the complete melting of the polymer, TiO_2_ powder was added. The mixing process lasted 30 min at the speed 30 min^−1^. The compound was manually taken out of the chamber and crushed using hand tools for the follow-up experiments.

### 2.5. Solid-State Polymerization

Fifteen samples of ST prepolymer were loaded into a specially designed tube furnace 1 m in length and equipped with a 6 kW power heater and a 3-channel temperature controller. In the first step, the temperature was gradually raised from room temperature to the target temperature (250 or 270 °C) over a period of approximately 1.5 h while maintaining a slow flow of argon. After heating for 6 h, the furnace was air-cooled to room temperature for about 30 min, and five samples were removed. During the cooling, inert atmosphere was preserved at least down to 150 °C. This procedure was repeated two additional times, resulting in sets of samples heated at the specified temperature for 6, 12, and 18 h, respectively.

### 2.6. Polymer Characterization

Randomly selected samples were weighed using Acculab ALC-210d4 laboratory analytical scales (New York, NY, USA) with a precision of four decimal places to control weight loss during solid-state polymerization. Density was determined using VIBRA AF-224RCE laboratory analytical scales VIBRA AF-224RCE (SHINKO DENSHI CO., LTD., Tokyo, Japan) equipped with a hydrostatic weighing attachment.

The measurement of logarithmic intrinsic viscosity ([*η*]) for the polymers was conducted at 60 ± 0.1 °C using an Ostwald viscometer with a 0.6 mm capillary and calculated using Equation (1):

(1) $$\left[\eta \right]=ln\left(t/t_{0}\right)/c$$

where *t* and *t*_0_ are the flow times of the polymer solution and the pure solvent through the capillary, respectively, while *c* is the concentration of the polymer solution (0.1 g of polymer per 100 g of pentafluorophenol).

Fourier-transform infrared (FTIR) spectroscopy was performed using an IFS-66 v/s IR-Fourier spectrometer (Bruker, Billerica, MA, USA) over a wavelength range of 4000–600 cm^−1^. The IR spectra were obtained in attenuated total reflectance (ATR) mode.

The liquid crystalline (LC) properties of the copolyesters were analyzed via polarizing optical microscopy (POM) on a 6 PO (Biomed, Moscow, Russia) device coupled with an FP900 thermal control system (Mettler, Columbus, OH, USA) and an E3ISPM5000 digital camera (ToupTek Photonics Co., Hangzhou, China). The temperature range of the measurements was 25–375 °C, with heating and cooling rates of 10 °C/min. A thin film of SI was prepared using hot pressing, while a thin film of ST was prepared through solution casting. Both films were heated to 350 °C and quickly cooled in air to room temperature.

The determination of Melt Flow Index (MFI) was conducted in accordance with the international standard [25] using the MFI measuring device IIRT-5 (Ivanovo, Russia) in the following conditions: *T* = 320 °C, a capillary with *d* = 2 mm and *L* = 8 mm, and weights of 2.16 and 21.6 kg applied to each sample.

The rotary rheometer RS 600 (Thermo HAAKE, Karlsruhe, Germany), equipped with a cone-plate unit (*d* = 20 mm, α = 2°), was used to maintain the measurements of complex shear modulus components as a function of temperature and oscillation frequency.

Strength, elongation, and elastic moduli were determined using a universal testing machine I-1140M (Tochpribor Ltd., Moscow, Russia) according to the international standard [24] with an extension rate of 10 mm/min.

The evaluation of thermal properties was conducted using Differential Scanning Calorimetry (DSC) and Thermogravimetric Analysis (TGA). Mass loss over time as the temperature changed was measured using the SKZ 1053A Thermogravimetric Analyzer (SKZ Industrial, Shizhong, Jinan, Shandong, China) under the following conditions: a heating rate of 20 K/min and an atmosphere of nitrogen. The DSC 204 F1 Phoenix device (NETZSCH, Selb, Germany) was employed to determine the glass transition according to the following protocol: samples weighing between 3.5 and 5.5 mg were heated in aluminum crucibles from 0 to 380 °C, then cooled to 0 °C, and subsequently reheated to 380 °C at a constant rate of 10 °C/min. The argon flow rate through the cell was set at 40 mL/min, while the protective gas flow rate of argon was maintained at 60 mL/min.

Thermomechanical analysis (TMA) was conducted using a TMA 402 F1 Hyperion device (NETZSCH, Selb, Germany) equipped with a compression measuring cell and a glass sample holder. A sample was clamped between two 1.5 mm-thick corundum discs. The sample had a parallelepiped shape with a contact area of 12.3 mm^2^. A sinusoidal load of 1 N ± 0.1 N was applied, and the heating rate was set to 3 °C/min in an inert argon (99.999% purity) atmosphere. A polymer sample was compressed into a disk at a temperature of 320 °C, with a pressure of less than 5 MPa applied to form it. The disks were then removed from the heated mold and allowed to cool to room temperature before being cut and flattened using a diamond blade. Subsequently, the resulting specimens were heated to 310 °C in an inert environment without any applied load to eliminate residual stresses. These specimens were then quenched in air for cooling.

## 3. Results and Discussion

### 3.1. Synthesis and Sample Preparation

**Figure 3:**
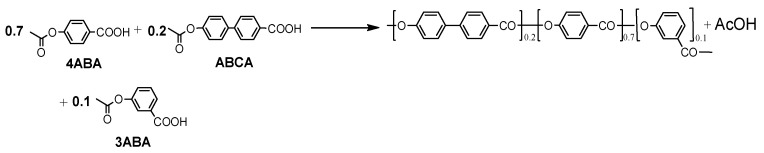
Synthesis of copolyester via melt polycondensation using 4-acetoxybenzoic acid (4ABA), 3-acetoxybenzoic acid (3ABA), and 4′-acetoxybiphenyl-4-carboxylic acid (ABCA).

Figure 3 illustrates the polymerization reaction using three acetoxycarboxylic acids—4ABA, 3ABA, and ABCA—in an inert atmosphere with molar ratios of 70%, 10%, and 20%, respectively. The method for producing fully aromatic homo- and copolyesters from acetoxycarboxylic (AB-type monomer) was proposed by researchers at Eastman Kodak in 1959 [26] and has been utilized, for example, in the synthesis of Vectra A-950 [15]. In the case of A-A + B-B type polycondensation, an equimolar mixture of diacetyl biphenol and dicarboxylic is employed as comonomers [27,28]. The acetoxycarboxylic acids and diacetyl biphenols can be generated in situ from the corresponding hydroxycarboxylic acids, biphenols, and an excess of acetic anhydride, which is advantageous for industrial production [18,20,22]. Both 4ABA and ABCA serve as mesogenic comonomers, while 3ABA, the meta isomer of 4ABA, disrupts the crystalline order in the copolymer. Poly(4-hydroxybenzoate) as well as poly(4′-hydroxybiphenyl-4-carboxylate) are intractable, with melting points exceeding their decomposition temperatures [29,30]. The lowest melting point recorded for the 4HBA:HBCA copolyester is 344 °C at a molar ratio of 70:30 [2,31]. Replacing 10 mol% of HBCA units with 3HBA units reduces the melting point to 315 °C [2]. Furthermore, molded articles produced from the copolyester with this composition exhibited the highest tensile strength.

The molded articles were produced through several steps. The growth of molecular weight in the samples during these steps was monitored using intrinsic viscosity (IV) measurements in pentafluorophenol (PFP) due to the insolubility of fully aromatic TMCPs in commonly used solvents. The first step involved molten-state polymerization, which resulted in a brittle product with low mechanical properties, referred to as the SI sample. A stainless-steel reactor, designed for high-viscosity media, was utilized for the synthesis of the SI sample. The reactor was connected to an argon line and a condenser to maintain an inert atmosphere. The synthesis was conducted over a period of 180 min, with gradual heating up to the apparatus limit of 273–274 °C. The molded article produced was brittle, and the IV measured as low as 0.5 (see Table 1). An additional solid-state polymerization (SSP) was performed for 6 h (2 h at 240 °C, 250 °C, and 260 °C, respectively) in a tube furnace under inert conditions, resulting in a prepolymer suitable for injection molding, designated as ST, with an IV of 3.1.

In the subsequent step, the prepolymer ST was molded into the articles. The SSP of these articles was conducted in a tube furnace, where they were maintained under a slow argon flow at specific temperatures of 250 °C and 270 °C, respectively. As shown in Table 1, the IV increases with an increase in SSP time. As the molecular weight of the copolyester increases, the complete solubility of the polymer in PFP diminishes. After 18 h of SSP at 250 °C and 12 h at 270 °C, the copolyester became insoluble in PFP.

**Figure 4:**
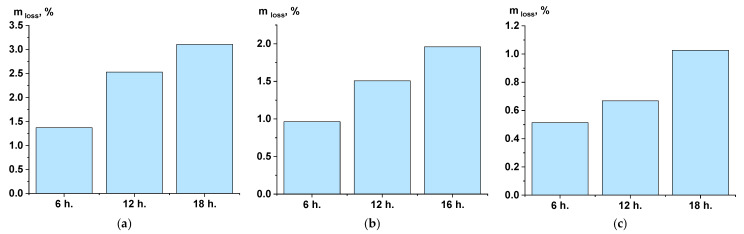
Weight loss of samples during solid-state polymerization ((**a**)—at 270 °C; (**b**)—at 250 °C; (**c**)—filled, at 250 °C).

The determination of mass loss showed that, during SSP at 270 °C, weight loss occurred more actively, as demonstrated by the histograms in Figure 4.

**Figure 5:**
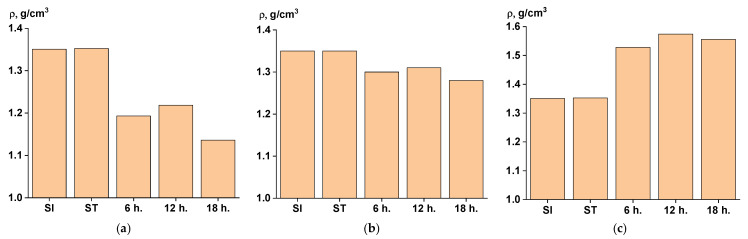
Density changing in dependence on time of solid-state polymerization ((**a**)—at 270 °C; (**b**)—at 250 °C; (**c**)—filled, at 250 °C).

At the same time, carrying out the process at 250 °C ensured a smoother change in the mass of samples from stage to stage, and hence a more uniform release of polycondensation products. This, in turn, affected the porosity of the samples, which can be judged by the change in density depending on the exposure time. This fact is confirmed by observing the dependence in Figure 5, which shows the change in density across different stages at various processing temperatures.

The addition of titanium dioxide, of course, increased the density of the material in comparison with the SI and ST samples, and it also eliminated the loss of mass and density in the composition at later stages of SSP in comparison with the samples (Figure 5a,b).

### 3.2. FTIR Spectroscopy

**Figure 6:**
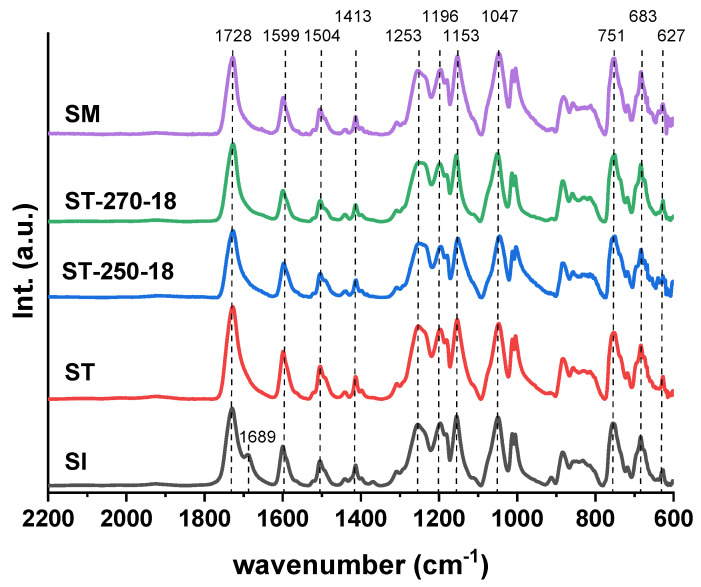
FTIR spectra of the samples.

Unlike the amorphous fully aromatic copolyesters and semiaromatic copolyesters, NMR analysis of TMFCCs presents significant challenges due to their limited solubility in solvents [10,14,32]. The samples SI, ST, ST-250-18, and ST-270-18 were analyzed using FTIR spectroscopy (Figure 6). For comparison, a spectrum of a copolyester (designated as SM) with the same composition described in previous work [2] was prepared by melt polymerization without SSP. No significant changes were observed in the spectra except for the spectrum of SI. A new band corresponding to the C=O vibration (*ν_C=O_*) of the ester group appeared at 1689 cm^−1^ for the acetic end group (CH_3_CO-). This indicates that SI is more akin to an oligomer than to a polymer. The spectra are very similar to those of the previously described ternary copolyesters based on 4HBA, HBCA, and vanillic acid, with a molar ratio of 3:1:1 [1].

### 3.3. Thermal Characterization

A comprehensive analysis of the thermal and thermomechanical properties was carried out to characterize the material as fully as possible and to identify the dependence of such properties on the temperature of the SSP, the duration of the process, and the addition of TiO_2_.

**Figure 7:**
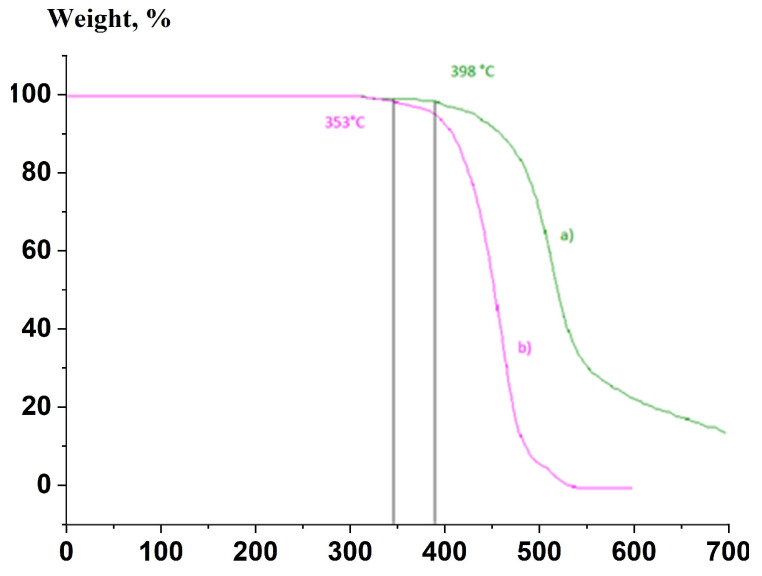
Mass loss curves: (**a**) sample ST-250-18; (**b**) sample ST-270-18.

TGA analysis demonstrated that the material was thermally stable up to 350 °C after 18 h of exposure at 270 °C, whereas exposing the SSP to lower temperatures allowed the creation of a more stable (up to almost 400 °C) material (Figure 7).

**Figure 8:**
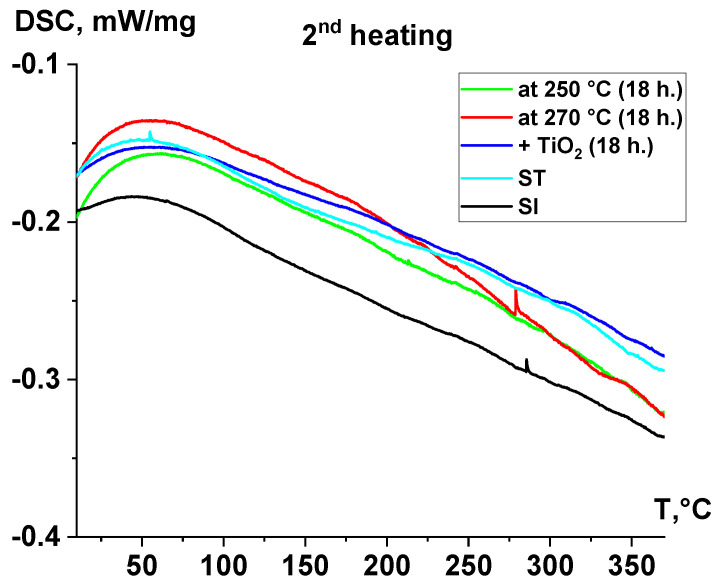
DSC curves of samples after second heating.

In the DSC curves of the samples (Figure 8), during the second heating cycle, there are no significant thermal effects associated with a vigorous reaction from solid-phase polymerization. However, the reaction continues to occur in all samples, which is reflected in the form of minor thermal effects, primarily observed above 200 °C. For all samples, there are no pronounced thermal effects on the DSC curves that can be attributed to the melting of the studied polymers. Above a temperature of 320 °C, there are no thermal effects indicative of an intensive thermal decomposition process, including in the sample filled with TiO_2._

**Figure 9:**
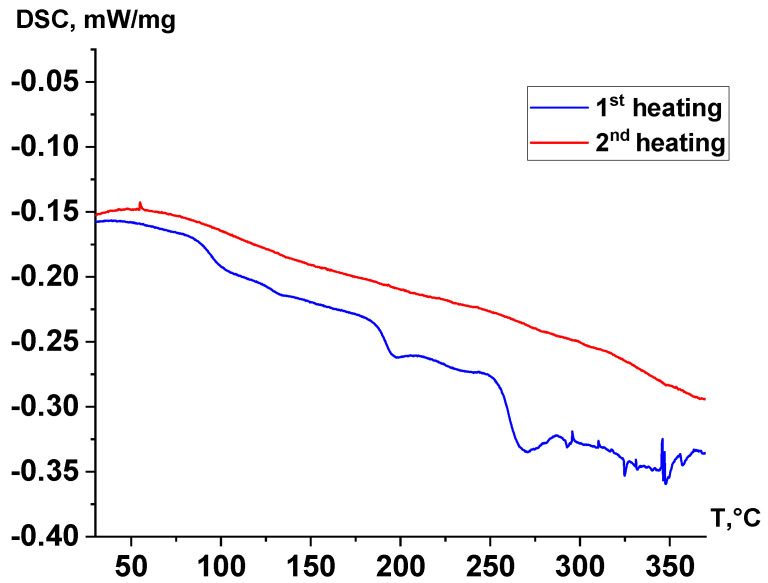
Comparison of DSC curves of sample ST after the first and second heating.

Figure 9 shows the DSC curves for the first (blue) and second (red) heating of the ST sample. Although the infrared Fourier spectra of the sample did not exhibit signals from acetic end groups, the molecular weight remained relatively low, indicating that polycondensation continued during heating. During the first heating, several endothermic effects were observed: the first two, occurring at approximately 95 °C and 195 °C, were likely associated with the melting of low-molecular-weight byproducts that accumulated in the sample during pre-annealing. The most pronounced minimum on the DSC curve, observed above 250 °C, corresponds to the onset of the polycondensation reaction, during which low-molecular-weight polycondensation products dissipate thermal energy. These effects were not observed during the second heating cycle. This can be attributed to the fact that, when a thin sample was loaded into a calorimeter and heated to 380 °C during the first cycle, it significantly increased its molecular weight to high values. In contrast, during the second heating cycle, the rate of polycondensation was considerably slower.

**Figure 10:**
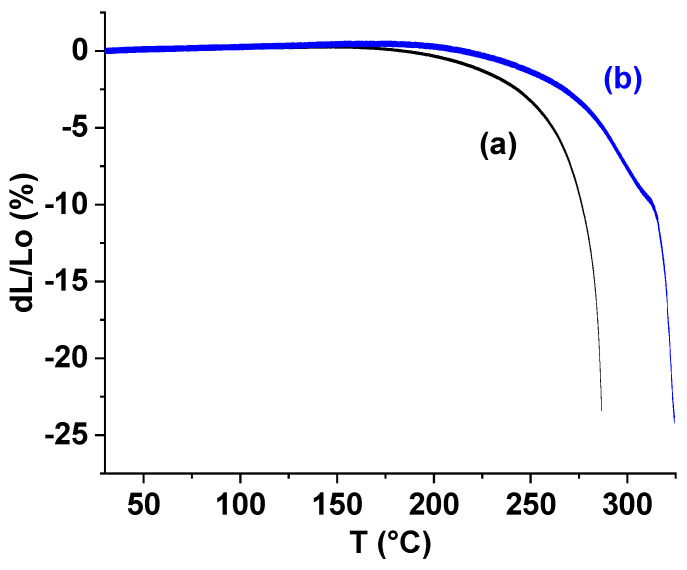
Thermomechanical curves of the sample ST-270-18 (a) and ST-270-18 + TiO_2_ (b).

**Figure 11:**
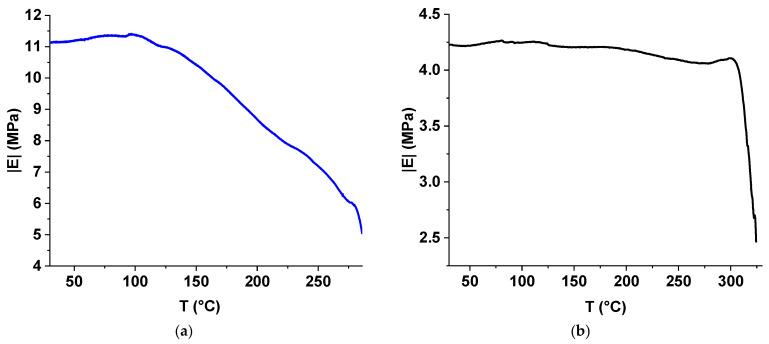
Dependence of Young’s modulus on temperature for sample ST-270-18 + TiO_2_ (**a**) and ST-270-18 (**b**).

**Figure 12:**
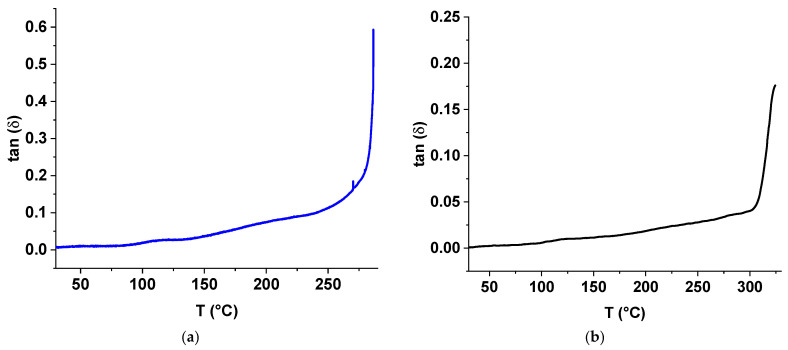
Dependence of the loss tangent on temperature for the samples ST-270-18 + TiO_2_ (**a**) and ST-270-18 (**b**).

The results of the TMA tests are shown in Figure 10, Figure 11 and Figure 12. A sample containing TiO_2_ was found to be more rigid than the unfilled sample; when subjected to the same cyclic loading, the sample with TiO_2_ deformed by 0.138% compared to 0.350% for the unfilled sample (see Figure 10). Above 172.7 °C, the ST-270-18 + TiO_2_ began to deform irreversibly (shrink), while the unfilled sample did not begin to deform until 190.4 °C. When the temperature reached 278.8 °C, both samples began to spread; however, the ST-270-18 + TiO_2_ did not fill until 312.2 °C.

The Young’s modulus of the unfilled sample remained stable up to the flow temperature. In contrast, the modulus of ST-270-18 + TiO_2_ decreased gradually above 105.9 °C, reducing by a factor of 1.8 before the flow occurred (Figure 11).

Filling the polymer with TiO_2_ increased its rigidity by 2.6 times, but worsened mechanical characteristics at temperatures above 106 °C and reduced the flow temperature under load by 33 °C.

### 3.4. Optical Spectroscopy

**Figure 13:**
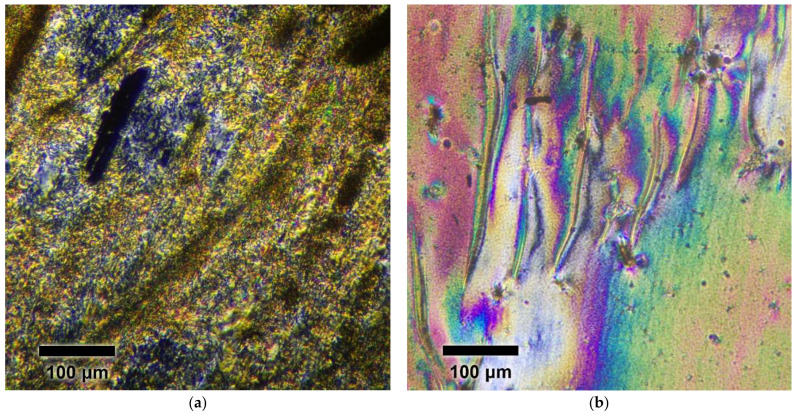
Polarized optical microscopy of quenched prepolymers heated from room temperature to 350 °C at 10 K/min: SI (**a**); ST 350 °C (**b**).

A thin film of low-viscosity oligomer SI was prepared by hot pressing between glass slides, while a thin film of prepolymer ST was obtained through solution casting of the ST solution in PFP onto a glass slide. Prior to optical observations, the films were heated to 350 °C and then quenched in air. The films exhibited textures characteristic of the liquid crystal (LC) phase after quenching. Heating both quenched samples to 375 °C and subsequent cooling them did not significantly alter the textures. No isotropization was observed for the SI and ST samples. Most fully aromatic TMCPs do not demonstrate a nematic-to-isotropic transition below 400 °C [6]. Figure 13 presents polarized optical microscopy images of the oligomer SI and prepolymer ST at 350 °C.

### 3.5. Rheological Properties

The rheological characteristics of the melting of a ternary fully aromatic copolymer are of particular interest because this material, when subject to complete polymerization, does not show flow even at temperatures above 300 degrees, which means that its processing, in particular by injection molding, is associated with high energy consumption and often impossible to perform. Carrying out SSP solves this problem, since it is proposed to use the ST prepolymer for molding items. It was proposed to determine the MFI of the ST sample, the value of which turned out to be equal to 93 g/10 min at a temperature of 320 °C and a load of 2.16 kg. For comparison, after the SSP the MFI was measured, it was shown that the MFI of the samples and the fluidity of the melt was significantly reduced, and in some cases, flow was almost impossible (see Table 2). This confirms the hypothesis that the ST prepolymer is the optimal material for casting such polymers. However, it should be noted that, in order to achieve the best quality of injection molding, it is necessary to degas the melt to remove bubbles, which inevitably form even under the conditions of a short injection cycle.

**Figure 14:**
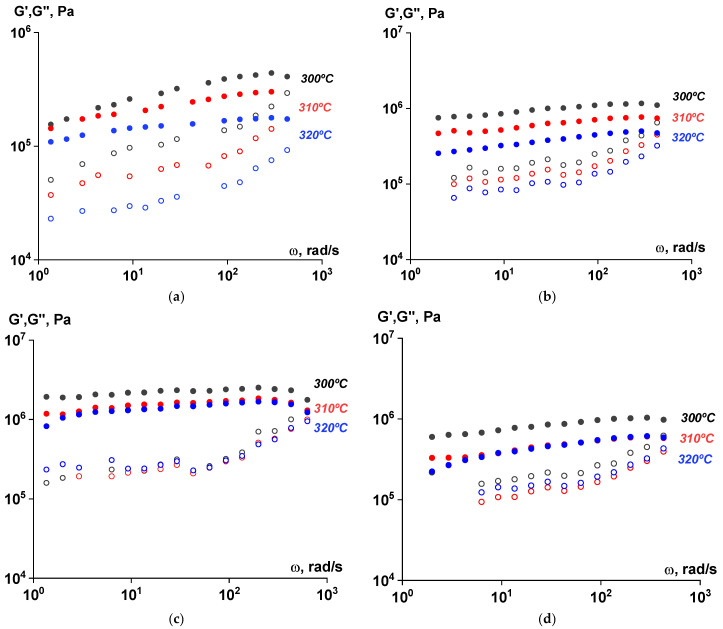
Frequency dependences of the components of the complex shear modulus (G′—filled symbols, G″—open symbols) for polymer samples subjected to solid-state polymerization at 270 °C at different temperatures. Amplitude of deformation of γ = 0.001 ((**a**)—ST polymer; (**b**)—after 6 h; (**c**)—after 12 h; (**d**)—after 18 h).

**Figure 15:**
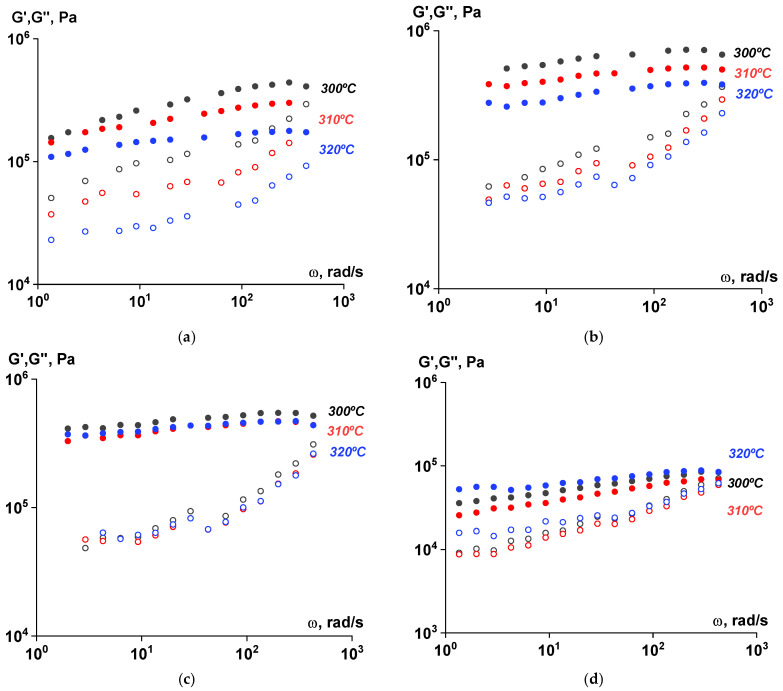
Frequency dependences of the components of the complex shear modulus (G′—filled symbols, G″—open symbols) for polymer samples subjected to solid-state polymerization at 250 °C at different temperatures. Amplitude of deformations of γ = 0.001 ((**a**)—ST polymer; (**b**)—after 6 h; (**c**)—after 12 h; (**d**)—after 18 h).

**Figure 16:**
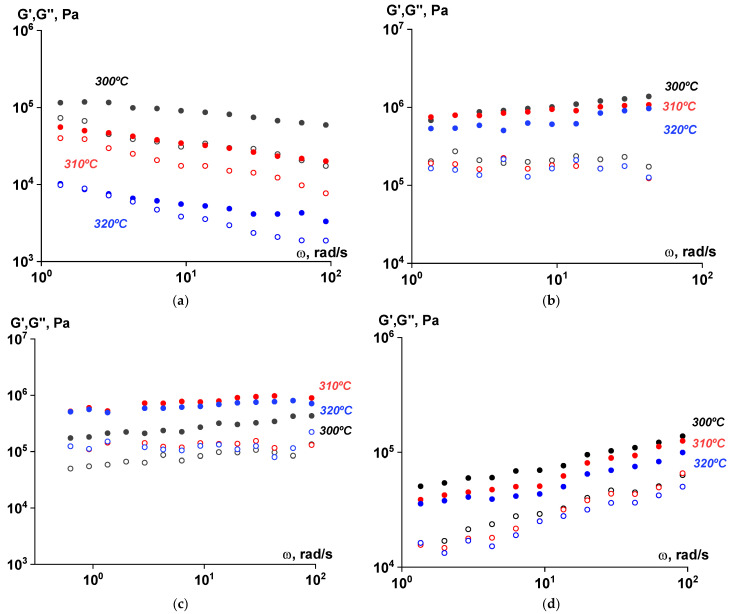
Frequency dependences of the components of the complex modulus (G′—filled symbols, G″—open symbols) for polymer samples filled with TiO_2_ subjected to solid-state polymerization at 250 °C at different temperatures. Amplitude of deformations of γ = 0.001 ((**a**)—before solid-state polymerization; (**b**)—after 6 h; (**c**)—after 12 h; (**d**)—after 18 h).

The viscoelastic characteristics of the samples at all stages of the SSP process were evaluated using rotational rheometry. As seen in Figure 14, Figure 15 and Figure 16, the values of the components of the complex dynamic modulus ranged from 10^4^ to 10^5^ Pa across the entire frequency range. The storage modulus values significantly exceeded those of the loss modulus, both at varying frequencies and temperatures. The dependences were linear, indicating that the material did not flow at the specified temperatures after the SSP and exhibited rather solid-like behavior.

### 3.6. Mechanical Properties

**Figure 17:**
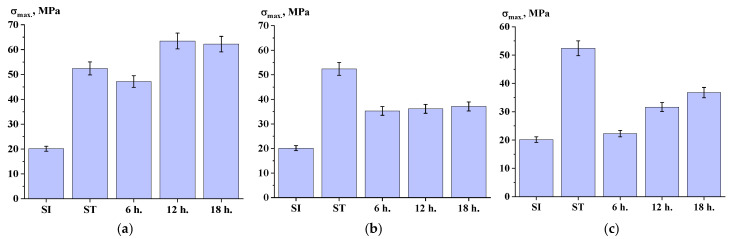
Ultimate tensile strength depending on time of solid-state polymerization ((**a**)—at 270 °C; (**b**)—at 250 °C; (**c**)—filled, at 250 °C).

**Figure 18:**
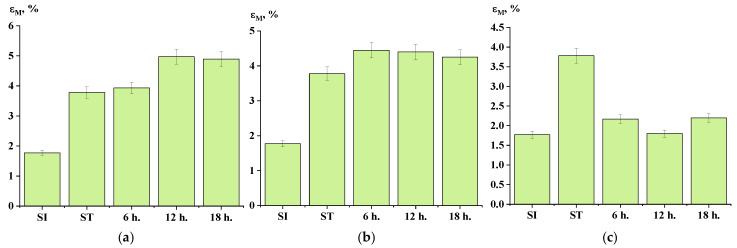
Elongation at break depending on time of solid-state polymerization ((**a**)—at 270 °C; (**b**)—at 250 °C; (**c**)—filled, at 250 °C).

**Figure 19:**
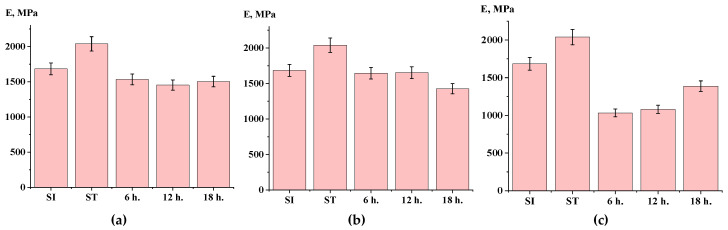
Elastic modulus depending on time of solid-state polymerization ((**a**)—at 270 °C; (**b**)—at 250 °C; (**c**)—filled, at 250 °C).

The data obtained from the measurement of mechanical properties indicate that conducting the SSP process at a temperature of 270 °C influences the strength of the material (Figure 17a). Overall, there was an increase in strength compared to the initial ST polymer. However, lowering the SSP temperature to 250 °C did not yield significant improvements in mechanical properties, nor did the addition of titanium dioxide at a concentration of 30 wt.%. As illustrated in Figure 18 and Figure 19, both the temperature of the SSP process and the presence of the filler did not have a substantial impact on the material’s elongation and modulus.

It can be stated that, to achieve the highest quality products using the SSP method, it is essential to ensure that the material is molded with minimal gas release during injection. Subsequently, the SSP process should be conducted at a temperature of no less than 270 °C, which will facilitate a more complete polymerization process, resulting in more durable products.

## 4. Conclusions

A mixture of 4ABA (70 mol%), 3ABA (10 mol%), and ABCA (15 mol%) underwent melt polycondensation through gradual heating to 273–274 °C in an inert atmosphere using a stainless-steel reactor. FTIR spectroscopy and capillary viscometry indicated that the reaction mixture from the reactor produced an oligomer with an IV as low as 0.5. The mixture was subjected to preliminary solid-state polycondensation for 6 h, resulting in the formation of a pre-polymer with an IV of 3.1, which was subsequently used to produce molded articles. The FTIR spectrum of the pre-polymer did not reveal any bands corresponding to acetic terminal groups. Polarized optical microscopy demonstrated that the oligomer and pre-polymer formed an LC phase. When heated to 375 °C, no transition to an isotropic state occurred, and no crystallization was observed upon cooling to room temperature. Following a relatively short solid-phase polycondensation, the oligomer transformed into a low-molecular-weight pre-polymer with low viscosity, facilitating processing via injection molding. The low viscosity of the pre-polymer enabled the preparation of molded articles and the blending of a compound from the pre-polymer and TiO_2_ at 320 °C using standard laboratory equipment. Molded articles were subsequently prepared from this compound. The subsequent SSP of the unfilled pre-polymer samples for 12–16 h at 250 and 270 °C significantly increased the molecular weight, resulting in an insoluble advanced polymer. SSP of the unfilled molded articles indicated that the molecular weight of the pre-polymer increased; however, polycondensation at 250 °C and 270 °C resulted in some porosity of the samples due to the release of low-molecular-weight byproducts, although this did not adversely affect the mechanical properties. To achieve products with optimal characteristics, it is essential to carefully select gradual heating protocols to prevent pore formation in the final products.

## Figures and Tables

**Table 1:** Conditions for solid-state polymerization and logarithmic intrinsic viscosity (IV) of the samples. (IV) of the samples.

Sample	SSP Temperature, °C	SSP duration, h	IV in PFP
SI	-	-	0.5
ST	-	-	3.0
ST-250-6	250	6	4.9
ST-250-12	250	12	6.3
ST-250-18	250	18	Insoluble
ST-270-6	270	6	4.5
ST-270-12	270	12	Insoluble
ST-270-18	270	18	Insoluble

**Table 2:** MFI of the selected samples.

Sample	T, °C	Load, kg	MFI, g/10 min
ST-270-18	320	2.16	0.4
ST-270-18	320	21.6	12.4
ST-250-18	320	2.16	5.7
ST-250-18	320	21.6	93
ST	320	2.16	102

## Data Availability

The original contributions presented in this study are included in the article. Further inquiries can be directed to the corresponding author.
